# Supplementary figures and images for: ScalaFlux: A scalable approach to quantify fluxes in metabolic subnetworks
Source: PLoS Comput Biol. 2020 Apr 14;16(4):e1007799. doi: 10.1371/journal.pcbi.1007799 (PMC7182278; doi:10.1371/journal.pcbi.1007799)

$^{13}\text{C}$ -enrichment

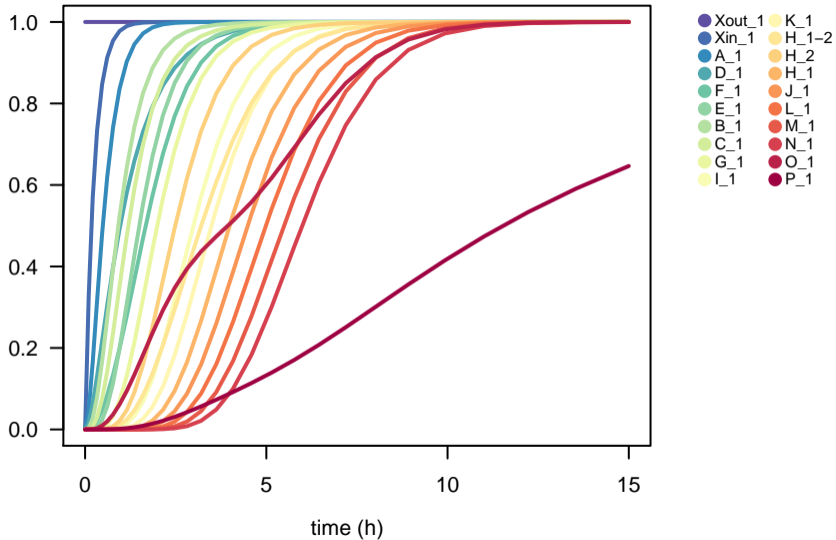

Supplement: S1 Fig — Simulated labeling dynamics of all metabolites of the example network (Fig 1A) in response to a switch from unlabeled Xout to fully labeled Xout, for fluxes and metabolite concentrations given in S1 Table. (PDF) [file pcbi.1007799.s002.pdf]

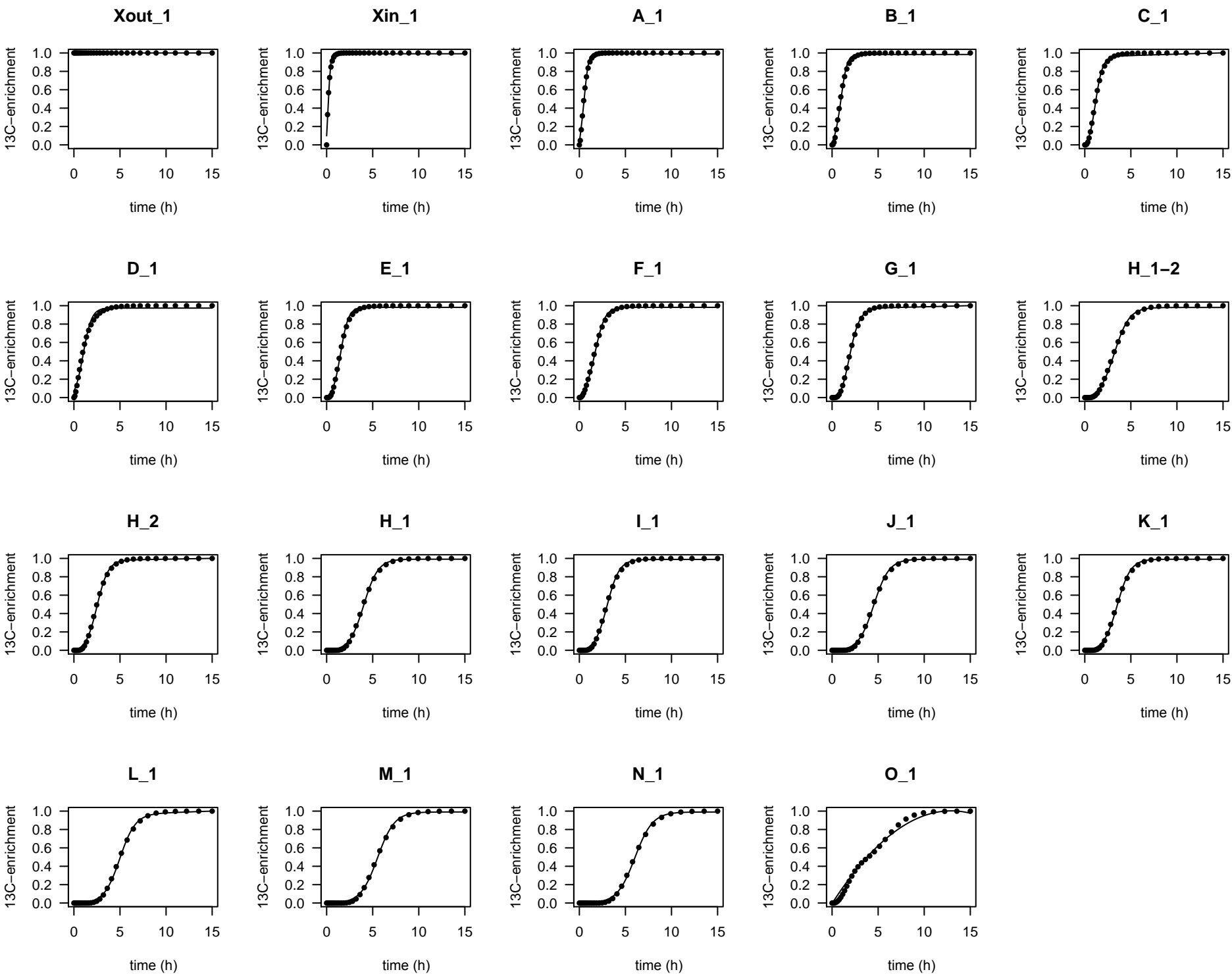

Supplement: S2 Fig — The labeling dynamics of the local label inputs of all the subsystems shown in Fig 3 were fitted with analytical functions (as detailed in the Methods section), based on the simulation results given in S1 Fig. The dots represent the fitted data and the lines represent the best fits. (PDF) [file pcbi.1007799.s003.pdf]

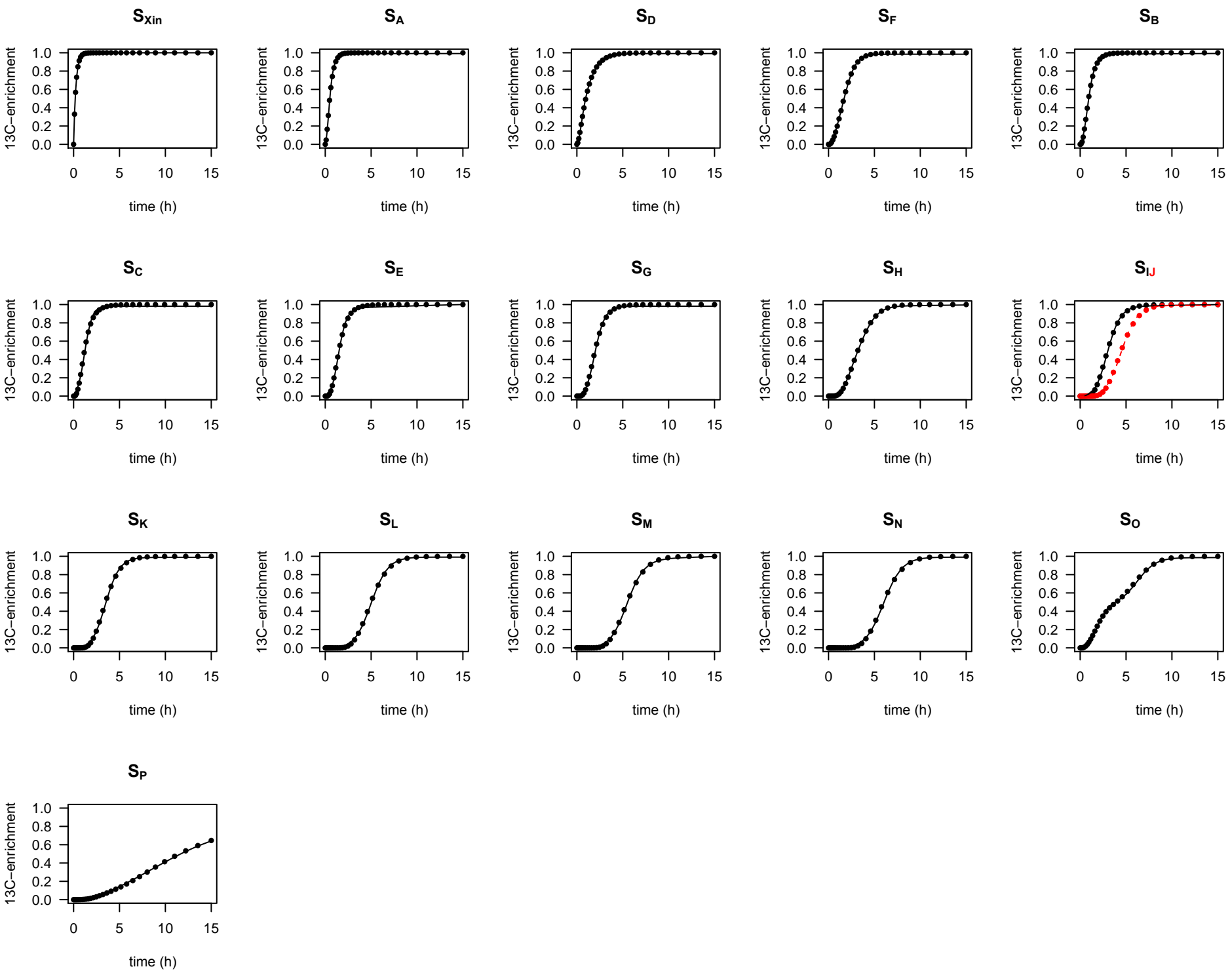

Supplement: S3 Fig — For all minimal subsystems of the example network (Fig 3), fluxes were estimated by fitting the labeling dynamics of the metabolic intermediate(s), using as (local) label input(s) the analytical functions obtained from the fits given in S2 Fig. For each subsystem, the dots represent the fitted data and the lines represent the best fits. The flux values and confidence intervals estimated from these fits are shown in Fig 3. (PDF) [file pcbi.1007799.s004.pdf]

**A)**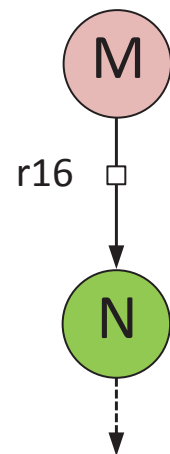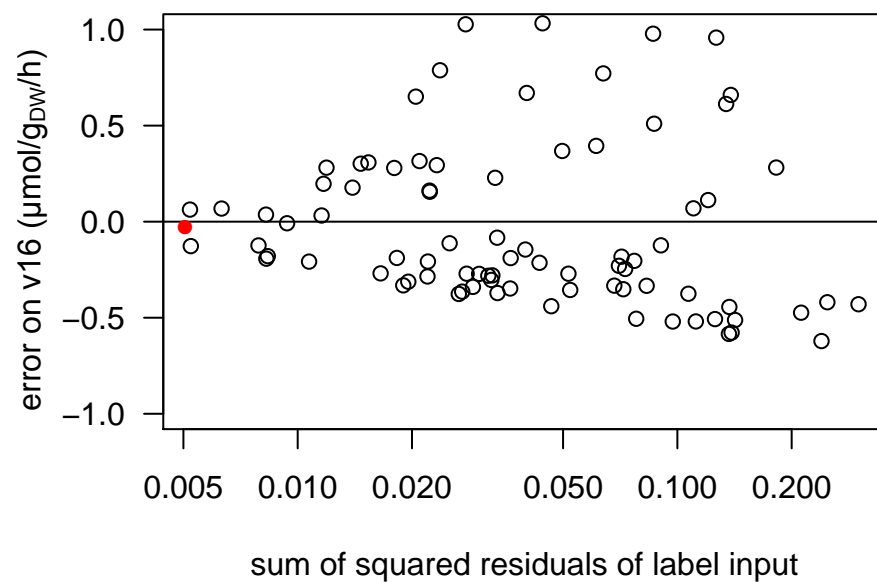**B)**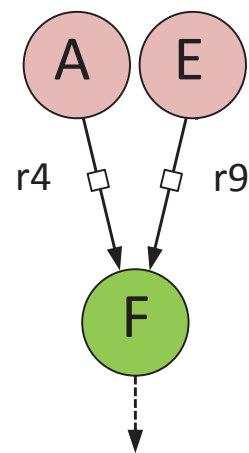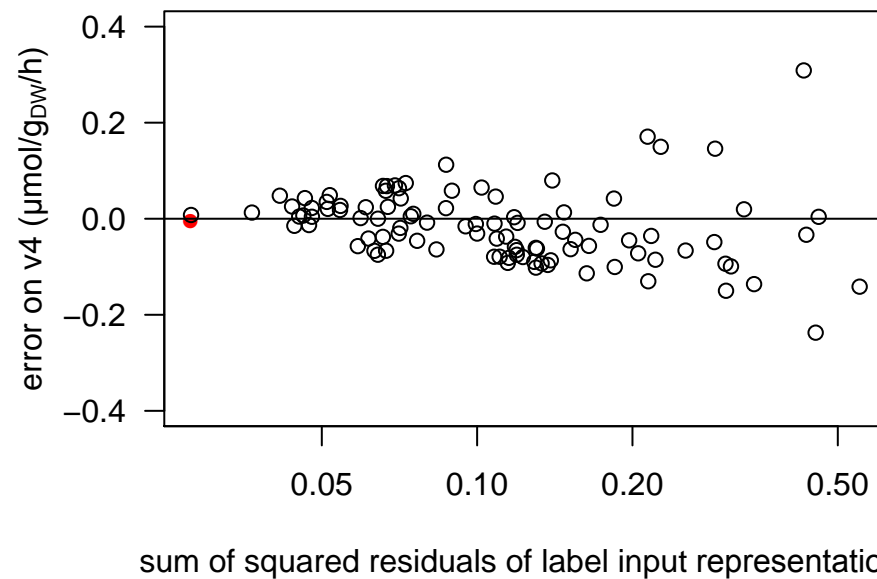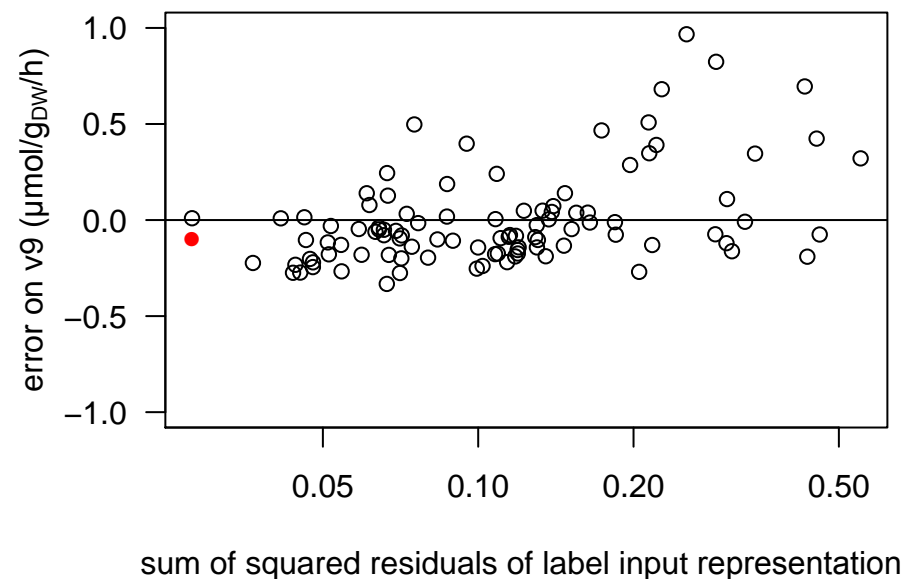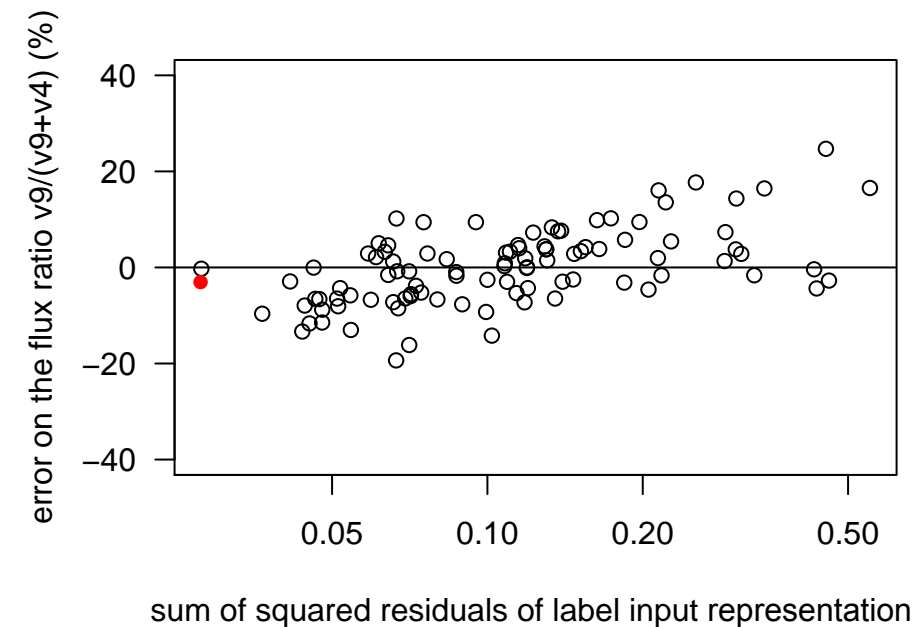

Supplement: S4 Fig — For two minimal subsystems (SN and SF in panels A and B, respectively), we degraded artificially the quality of the fit of label inputs by varying parameters of the analytical functions (100 sets of parameters were randomly sampled within ± 5% of their optimal values), and we calculated how the fluxes estimated from the degraded analytical functions deviate from the true values. Plots show the error on the estimated fluxes (and on the relative contribution of the two converging reactions for SF) as function of the error on the representation of label inputs (sum of squared residuals for the degraded analytical functions of label inputs). The red dots represent results for the best fits (i.e. with parameters of the analytical functions set to their optimal values). (PDF) [file pcbi.1007799.s005.pdf]
