# Supplementary material for: ScalaFlux: A scalable approach to quantify fluxes in metabolic subnetworks
Source: PLoS Comput Biol. 2020 Apr 14;16(4):e1007799. doi: 10.1371/journal.pcbi.1007799 (PMC7182278; doi:10.1371/journal.pcbi.1007799)
Supplement: Attachment — Submitted filename: NA.docx [file pcbi.1007799.s006.docx]

We have modified the manuscript according to the technical changes you have requested.
