# Supplementary material for: ScalaFlux: A scalable approach to quantify fluxes in metabolic subnetworks
Source: PLoS Comput Biol. 2020 Apr 14;16(4):e1007799. doi: 10.1371/journal.pcbi.1007799 (PMC7182278; doi:10.1371/journal.pcbi.1007799)
Supplement: Attachment — Submitted filename: Scalaflux_Response_to_reviewers.docx [file pcbi.1007799.s007.docx]

Reviewers comments’ are in **black**, our responses are in **blue.**

**Reviewer #1:** The authors present a novel method for 13C-flux analysis with clear advantages over current approaches. By defining suitable subnetworks, flux estimation is possible with ScalaFlux for pathways not accessible with current methods aiming for holistic flux fitting. While I find the advantages very important and interesting and the method generally well presented, I think that some issues require clarification. Please, find my detailed concerns below.

We thank reviewer 1 for his/her positive feedback and for his/her relevant suggestions, which were valuable for improving the manuscript. We address all his/her concerns and respond point by point to his/her comments below.

1. The authors state that ScalaFlux has been implemented on top of previously developed methods in software IsoSim. It remains somewhat unclear what are the novel parts that are proposed here for publication. Please, clarify the novelty compared to previous methods in IsoSim.

In the revised manuscript, we have clarified the novel parts of IsoSim that were specifically developed to implement the ScalaFlux approach. These novel parts, which focus on label input(s), consist in: i) fitting analytical functions to experimental labelling dynamics of the (local) label inputs and ii) use time-dependent functions as label input to model isotope propagation (see the revised Methods section). These novel parts are necessary and sufficient to allow the conceptual change brought by ScalaFlux, which is why we believe that “*this approach can be implemented in existing ^13^C-flux calculation software with minimal efforts*” (line 420 of the original manuscript). In fact, besides having implemented ScalaFlux in IsoSim as proof of concept, we are also currently implementing the ScalaFlux approach in *influx* (<https://github.com/sgsokol/influx/> , Sokol *et al.*, 2012, DOI: 10.1093/bioinformatics/btr716), a more mature and robust software for stationary and instationary ^13^C-metabolic flux analysis. We now detail this point in the Discussion:

“

Indeed, the key features required to implement ScalaFlux in existing software focus on the definition of label input(s). These features, which are necessary and sufficient to allow the conceptual change brought by ScalaFlux, consist in fitting analytical functions to experimental labeling dynamics of the (local) label inputs, and in using time-dependent functions as label input to model isotope propagation.

“

2. It remains also not clearly stated, for what kind of growth conditions is the ScalaFlux method suitable to. It should be clearly stated that metabolic (pseudo) steady state is required.

We agree with this comment and have made this point explicit in the revised manuscript.

3. It does not also become clear how the limitations of mean molecular enrichment data are reflected in the flux identifiability or unidentifiability in specific kinds of subnetworks involving cleavage.

It is important to stress that ScalaFlux is not limited to the exploitation of mean molecular enrichment data but can be used to calculate fluxes from any type of measurements (specific enrichments and/or positional isotopomers collected by NMR, as well as isotopologue distributions of metabolites and/or fragments collected by MS and MS/MS) and any network topology. The limitations with respect to the type of measurements used to calculate fluxes are now detailed in the revised manuscript for different kinds of subnetworks (including subnetworks involving cleavage reactions). To clarify this important question, we have added the following paragraph in the section “*Flux calculation in minimal metabolic subsystems*” (these considerations are equally valid for combined subsystems):

“

To ensure flux identifiability, the isotopic information collected on a given (sub)set of carbon atom(s) of the label input(s) must match the isotopic information collected on the corresponding (sub)set of atom(s) of the product(s). For the simplest minimal subsystem containing a single unimolecular reaction (e.g. *S_A_*), the carbon atoms of the label input (*X_in_*) directly matches the carbon atoms of the product (*A*). The flux (r2) can thus be calculated from global information collected on each metabolite (isotopologue distributions or mean molecular enrichments measured by MS) as well as from positional information on matching atoms (specific enrichments or positional isotopomers measured by NMR). For minimal subsystems involving condensation reactions (e.g. *S_H_*), the flux (r11) can be estimated from global measurements of the labeling dynamics of the two label inputs (*G* and *K*) and of the product (*H*). The labeling dynamics of only one of the two label inputs is sufficient for flux calculation when the labeling of the corresponding subset of carbon atoms of the product is available (e.g. when a fragment containing these atoms can be measured by MS/MS, or when positional information on matching atoms are accessible). Finally, for minimal subsystems where the product is formed by a cleavage reaction (e.g. *S_I_*), global isotopic information may be collected on the product (*I*), and the corresponding information must be measured on the matching fragment of the local label input (*H*). If no matching fragment is available, fluxes may still be calculated using positional information collected by NMR. As an alternative strategy, identifiability may also be enhanced by combining minimal subsystems (see section “*From individual reactions to metabolic pathways: combining minimal subsystems enhances flux identifiability and precision*”).

“

4. In addition, it remains unclear how does the shape of the function fitted to convert the discrete data into continuous affect the flux estimation.

In the revised manuscript, we analyse in detail how the shape affect the flux estimation. We artificially degraded the quality of the fit of local label inputs (by changing parameters values of the analytical functions), and we analysed the relationship between the error on estimated fluxes and the degree of degradation of the quality of the representation of local label inputs. As intuitively expected, the error on the estimated fluxes increased with the error on label inputs representation.

In the current version of IsoSim, we provide a fitting function typically used by the community to fit labelling dynamics (logistic function as implemented for instance in Dynamet, DOI: 10.1021/acs.analchem.5b01660) but also another function that may fit more complex labelling profiles (modified double logistic function proposed by Elmore et al., DOI: 10.1111/j.1365-2486.2011.02521.x). If the shape of these functions cannot fit accurately the experimental labelling dynamics of label inputs, users may implement their own functions in IsoSim. The simulation routines of IsoSim accepts any time-dependent analytical function as label input, which gives users the possibility to test how the shape of the function impact their own flux calculations. In this manuscript, we demonstrate that the functions implemented in the current version of IsoSim provide accurate estimates of flux values both on theoretical and on real situations (Figures 3, 4 and 5), at least for the networks and datasets considered here.

To clarify this point, we have added a new paragraph in the revised manuscript:

“

We tested how the shape of the functions that represent local label inputs affect the fluxes by degrading artificially the quality of the fit of label inputs. We varied parameters of the analytical functions (parameters were sampled randomly within ± 5 % of their optimal values), and we analyzed the distribution of errors on the estimated fluxes as function of the error introduced on the representation of label inputs (S4 Fig). We carried out this analysis on two minimal subsystems composed of a single reaction (*S_N_*) or of two converging reactions (*S_F_*). For the minimal subsystem *S_N_*, the error on the estimated flux r16 was minimal for parameters corresponding to the best fit of label inputs, and the error increased when the fit was degraded (S4 Fig). The same phenomenon was observed in *S_F_*, both for individual fluxes (r4 and r9) and for the relative contribution of each reaction to the biosynthesis of F (S4 Fig). The flux sensitivity depends on the subsystem, on the reaction (e.g. the flux r16 in *S_N_* is more sensitive against parameter variation than the flux r4 or r9 in *S_F_*), and on the inferred information (e.g. for *S_F_*, the flux ratio seems to be less sensitive against parameter variation than the flux r9). These results stress the need of providing accurate representations of label inputs for flux calculation. If a given analytical function cannot fit accurately the experimental labeling dynamics of some label inputs, one should therefore find another function that better represents label inputs, and test how the shape of this function impact their own flux calculations.

“

5. In Introduction on line 70 it is stated that the intracellular concentrations of metabolites would need to be measured for flux identifiability, but this of course does not hold for many steady state 13C-flux analysis methods as the above in the Introduction mentioned metabolic flux ratio analysis.

We agree with this comment. In the revised manuscript, we have rephrased the introduction to indicate that the intracellular concentrations of metabolites would need to be measured to ensure flux identifiability only for instationary ^13^C-metabolic flux analysis.

6. Flux units are missing from page 12 and from figures 3 and 4.

We have added flux units in the revised manuscript.

**Reviewer #2:** Major
The approach was mostly demonstrated over an example network. The real system used is rather a simple linear-like pathway. I believe the approach should be applied on one other real system with a larger pathway to better demonstrate its applicability.

As highlighted in the manuscript and noted by reviewer 1, the main value of our approach is specifically to be able to focus on a specific pathway of interest, and thereby to address a targeted biological question while reducing both the analytical and computational burden that arise with classical approaches where we have to deal with large networks (and datasets). One of the main benefits of ScalaFlux is thus that we have to deal with neither large networks nor the major constraint in ^13^C-MFA to include all steps - and labelling data - in between the label input and the (set of) reaction(s) of interest. We agree that the real system used here is a “*simple linear-like pathway*”, but none of the existing approaches are able to estimate fluxes through this pathway, as “*simple*” as it is. From our point of view, this validates both the scalability and the applicability of ScalaFlux. We believe that the extensive theoretical and practical demonstration of the scalability of ScalaFlux, using an example network as well as a real system (and real data from three different strains), is sufficient to validate this approach. We do not see any benefit of applying ScalaFlux to one other real system with a larger network, which we are already able to cope with using existing software developed by us (influx) or others (e.g. 13CFlux2, INCA, etc).

Minor
1) The abbreviation EMU is not defined in the manuscript. Please shortly define it.

We have defined this abbreviation in the revised manuscript.

2) The pipeline in Figure 1 includes sensitivity (Monte Carlo analysis). However this step is never defined in the manuscript. To ensure thoroughness of the paper it should shortly be defined/explained in the manuscript.

To ensure thoroughness of the paper, we have added a brief explanation of the Monte Carlo sensitivity analysis in the Methods section, and we now refer to Monte Carlo analysis in the Results section.

3) In Figure 1, D is formed from Xin, but in Fig.2 and Fig.3 It is typed as Sin, not Xin. Please correct. Similarly, in Supplementary Table, I see Sin and Sout, not defined in Figure 1. Probably they should be Xin and Xout. Also, the flux v6xch in the supplementary table is not defined in Figure 1.

We thank reviewer 2 for spotting these typos, which have been corrected in the revised manuscript.

4) Supplementary figures are a bit confusing .Please give more information in the legends of supplementary figures. It also looks supplementary figure labels are mixed. In supplementary figure S1, what do the figure titles (S1, S4_9 etc) correspond to? And why do we see a red and black curve in S12 in this figure?

We have added detailed information in the legends of Supporting figures. We have changed the titles of each graph of Supporting Figure S3 to make the notation consistent with the rest of the manuscript. Fluxes through S_I_ and S_J_ are calculated based on the same flux model (S_I_ and S_J_ contain the same reaction, as shown in Figure 2A) by fitting the labelling dynamics of the two products (I and J), which is why there is a red and black curves in this plot.

5) Regarding Figure 3A and Supplementary Table 1, Is there any specific reason behind selecting and specifying those five fluxes out of all fluxes?

These five fluxes represent the degrees of freedom of the system under metabolic steady-state, i.e. knowing their values is sufficient to recalculate all other fluxes. For sake of clarity, we now specify all fluxes in the revised Supporting information (S1 Table).

6) The following explanation in line225-226 is not clear: “assuming a typical precision 0.02 for 13C-enrichments and of 10 % for concentrations”. What is precision? Why is one defined as absolute value and the other one as a percentage?

We now used “relative standard deviation” rather than “precision”. We rephrased this sentence by “assuming a typical relative standard deviation of 2 % for ^13^C-enrichments and of 10 % for concentrations”.
